# Age Group-Specific Assessment of Changing Seroepidemiology of Hepatitis A Virus Infection in North India

**DOI:** 10.7759/cureus.30792

**Published:** 2022-10-28

**Authors:** Jyotsana Agarwal, Sugandha Srivastava, Bhanu P Verma, Palak Mehrotra

**Affiliations:** 1 Microbiology, Dr. Ram Manohar Lohia Institute of Medical Sciences, Lucknow, IND

**Keywords:** viral hepatitis a, anti-hav igg antibodies, hepatitis a vaccination, seropositive, hepatitis a virus serology

## Abstract

Introduction

Routine immunization against hepatitis A virus (HAV) infection has not been warranted in India, but an epidemiological shift from hyperendemicity to intermediate endemicity has been detected in recent years. The present study was planned to gather the age group-specific seroprevalence data of hepatitis A IgG antibodies in various age groups and evaluate any early trends of seroepidemiological shift.

Method

This was a hospital-based cross-sectional study. The detection of IgG antibodies for hepatitis A was done using an HAV Ab kit (Dia.Pro, Milan, Italy) in sera of individuals from >1 to 80 years of age and consenting to participate. Data on sociodemographic factors and potentially predisposing factors of HAV was collected on a predesigned questionnaire. At the time of final analysis, patients were divided into three groups children one to <18 years, adults ≥18 to <60 years, and old ≥60 to 80 years for comparative analysis.

Result

A total of 1,250 patients were included in the final analysis (129 children, 928 adults, and 193 old). The male/female ratio of the study participants was 1.4:1. The majority (85%) of them came from rural and semi-urban areas. They generally had lower socioeconomic status (SES) with poor literacy rates. Most of the enrolled cases (n=800/1,250, 64%) reported the use of groundwater, and 58.7% (n=734/1,250) consume water without any purification. Of the study participants, 90.8% reported the use of toilets for defecation, and 96.7% of the cases use soap for handwashing after defecation. The majority of adult (90%) and old age (99%) participants were seropositive for anti-HAV IgG antibodies as compared to children (80%). No significant differences were observed in the seropositivity rates and the SES class of the study participants.

Conclusion

About 20% of children did not have anti-HAV IgG antibodies in the present study, indicating that they are not exposed to HAV. This could be because of their better living conditions such as the availability of safe drinking water and improved sanitation and hygiene. We support the current guidelines of the Indian Academy of Pediatrics (IAP), which recommends immunization for hepatitis A vaccination at 12 months of age. Adult vaccination is not needed in North India.

## Introduction

Hepatitis A virus (HAV) infection is an acute, self-limiting disease of the liver transmitted via the fecal-oral route. India is hyperendemic for HAV infection [1]. The disease is closely associated with a lack of safe water, inadequate sanitation, and poor personal hygiene. The clinical spectrum of HAV varies from asymptomatic infection in children to fulminant and potentially fatal hepatitis in adults [2]. Routine immunization against HAV has not been mandated in India, and it was always believed that by the end of the first decade of life, most population develops antibodies against HAV and therefore is not at risk of infection [2]. However, in recent studies, an epidemiological transition from hyperendemicity to intermediate endemicity has been observed possibly due to rapid improvement in socioeconomic status (SES) in developing countries in recent years [1,2]. The HAV antibody seroprevalence rates in India are the lowest in Kerala in 4%-5% of the population, presumably because of better living conditions [3]. Local data, however, from Uttar Pradesh and neighboring areas are still missing. The Indian Academy of Pediatrics (IAP) 2016 Immunization Schedule recommends hepatitis A vaccination at 12 months of age, but this is still not a part of the essential routine vaccination program [4]. Before including hepatitis A vaccine in the routine vaccination schedule, it will be useful to have sufficient epidemiological data as it imposes a huge economic burden on the country too. Therefore, the present study aimed to gather the age group-specific seroprevalence data of hepatitis A IgG antibodies in various age groups and evaluate any early signs of the seroepidemiological shift in HAV infection and accordingly propose appropriate strategies for vaccination for specific susceptible age groups upon their proper characterization.

## Materials and methods

Study design and subjects

This was a hospital-based cross-sectional study. Participants were enrolled from blood donors of blood bank and patients attending the medicine/pediatric outpatient department (OPD) of Dr. Ram Manohar Lohia Institute of Medical Sciences, Lucknow, India, for appropriate age brackets after obtaining written informed consent. All individuals of first, second, third, fourth, fifth, sixth, seventh, and eighth decades, i.e., from one to 80 years of age, and willing to participate were invited for the present study. Participant exclusion criteria included a previous history of hepatitis A vaccination or past history of jaundice. The study protocol was approved by the institutional ethics committee (Reference Number: IEC 21/17).

Data collection

Data on sociodemographic factors and potential predisposing factors of HAV were collected on a standardized questionnaire for each participant. Information on education, occupation (occupation of the family head, in case of a minor), and monthly family income were used to calculate socioeconomic status (SES) as per revised guidelines of the Kuppuswamy Socioeconomic Scale 2017 [5].

Sample size

The sample size was estimated on the prevalence of the “absence of IgG antibodies for HAV,” i.e., 1.25% (based on a pilot study on 80 healthy individuals), at a 95% confidence interval and margin of error of 0.625% of the given prevalence; the calculated sample size came out to be 1,214. The sample size was estimated using Power Analysis and Sample Size version 8 (PASS-2008) (NCSS, LLC, Kaysville, UT, USA).

The detection of IgG antibodies for hepatitis A was done using the HAV Ab kit (Dia.Pro, Milan, Italy) in sera samples. About 1 mL of blood samples will be taken in separate plain vials when venepuncture was done to collect blood for routine investigations The sera were separated, aliquoted, and stored at -20°C until tested using enzyme-linked immunosorbent assay (ELISA) in the Department of Microbiology. Pre-assay controls were tested, and test results were verified as per the manufacturer’s instructions by means of the cutoff value determined. A person was considered to have protective antibodies if ELISA for IgG antibodies to HAV tested positive in their sera.

Data management and analysis

Data were entered in a Microsoft Excel sheet (Microsoft Corp., Redmond, WA, USA). For final data cleaning, the whole data was imported into the Statistical Package for the Social Sciences software version 23 (IBM SPSS Statistics, Armonk, NY, USA). A total of 1,250 study participants were included in the final analysis. At the time of final analysis, the patients were divided into three groups, children one to <18 years, adults ≥18 to <60 years, and old ≥60 to 80 years, for comparative analysis. The descriptive statistics for various variables were reported as frequency and percentage for qualitative variables. A P value < 0.05 was considered statistically significant.

## Results

A total of 1,765 patients were screened to check their eligibility for participation in the current study. Of these patients, 515 were excluded due to a prior history of jaundice (n=467/515, 90.7%) or vaccination for HAV (n=48/515, 9.3%). Thus, a total of 1,250 patients were finally included in the present study. Of these, 129 participants were children, i.e., one to <18 years old; 928 were adults, i.e., ≥18 to <60 years; and 193 were old aged, i.e., ≥60 to 80 years. The male/female ratio of the study participants was 1.4:1. The age decade-wise detailed gender distribution of the participants is shown in Table 1.

**Table 1:** Gender-wise distribution of the study participants (N=1,250)

Study participants	Age bracket (years)	Total number	Male	Female	Male/female ratio
Children (1 to <18 years) (N=129)	First decade (1-10)	52	35	17	2.1:1
	Second decade (11-20)	77	48	29	1.7:1
Adult (≥18 to <60 years) (N=928)	Third decade (21-30)	216	120	96	1.3:1
	Fourth decade (31-40)	215	96	123	0.78:1
	Fifth decade (41-50)	236	135	101	1.3:1
	Sixth decade (51-60)	261	163	98	1.7:1
Old (≥60 to 80 years) (N=193)	Seventh decade (61-70)	139	96	43	2.2:1
	Eighth decade (71-80)	54	39	15	2.6:1
Total		1,250	732	518	1.4:1

The majority (85%) of patients attending the hospital came from rural and semi-urban areas and belonged to lower socioeconomic strata with poor literacy rates. The data of study variables noted for each participant on a predesigned questionnaire was used to calculate the SES status (Table 2).

**Table 2:** Demographic characteristics of the study subjects (N=1,250) *Note: Data shown in parenthesis are % values.

Variables	Children (1 to <18 years) (N=129)	Adult (≥18 to <60 years) (N=928)	Old (≥60 to 80 years) (N=193)
Education score			
Profession or honors	0	9 (0.96 )	0
Graduate or postgraduate	0	178 (19.18)	9 (4.6)
Intermediate or post-high school diploma	10 (7.75 )	193 (20.79)	38 (19.68)
High school certificate	16 (12.40 )	187 (20.15)	42 (21.76)
Middle school certificate	38 ( 29.45)	216 (23.27)	46 (23.83)
Primary school certificate	51 (39.53)	98 (10.56)	28 (14.50)
Illiterate	14 (10.85)	47 (5.06)	30 (15.54)
Occupation			
Profession	0	7 (0.75)	0
Semiprofessional	0	125 (13.46)	7 (3.6)
Clerical, shop owner, farmer	24 (18.60)	367 (39.54)	88 (45.59)
Skilled worker	0	293 (31.57)	48 (24.87)
Semiskilled worker	0	98 (10.56)	8 (4.14)
Unskilled worker	12 (9.30)	0	0
Unemployed	93 (72.09)	38 (4.09)	42 (21.76)
Family income per month (in rupees)			
41,430-43,000	0	6 (0.64)	0
20,715‑41,429	0	103 (11.09)	7 (3.62)
15,536‑20,714	38 (29.45)	334 (35.99)	73 (37.82)
10,357‑15,535	56 (43.41)	227 (24.46)	83 (43)
6,214‑10,356	20 (15.50)	191 (20.58)	26 (13.47)
2,092‑6,213	11 (8.5)	57 (6.14)	4 (2.07)
<2,091	4 (3.10)	10 (1.07)	0
Socioeconomic class			
Upper class	0	33 (3.55)	1 (0.51)
Upper middle class	37 (28.68)	248 (26.72)	28 (14.50)
Lower middle class	89 (68.99)	439 (47.30)	141 (73.05)
Upper lower class	3 (2.32)	170 (18.31)	19 (9.84)
Lower class	0	38 (4.09)	4 (2.07)

**Figure 1:**
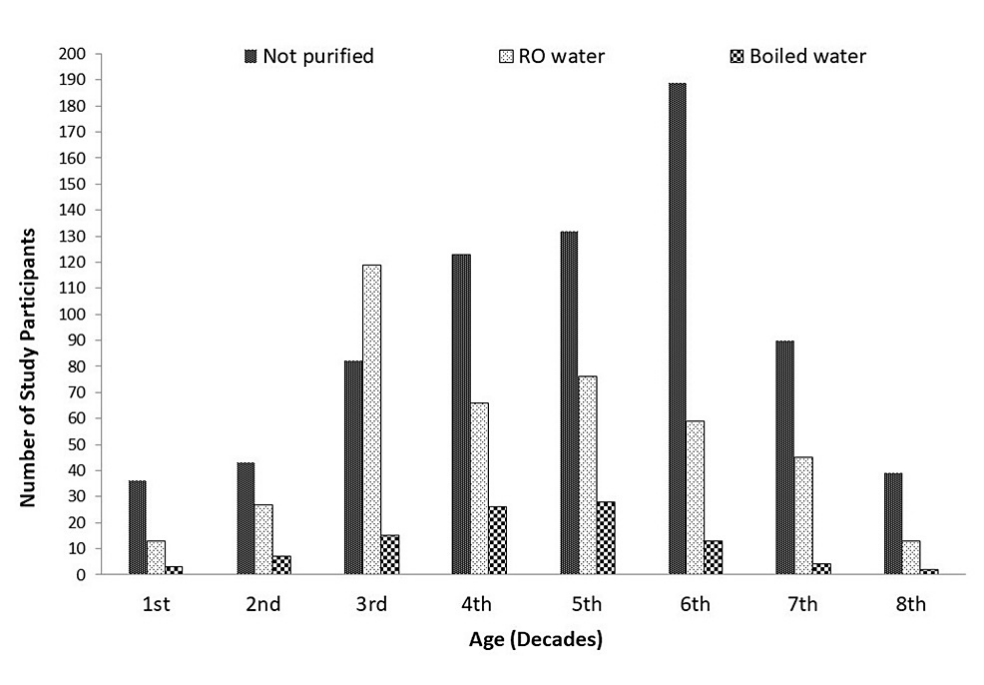
Use of water purification method by various age decades of the study participants RO water: reverse osmosis method of water purification

Of the 1,250 patients enrolled, the majority (n=800, 64%) reported the use of groundwater, 58.7% (n=734) of the participants consumed water without any purification, 33.4% (n=418) used the reverse osmosis method of water purification, and 7.9% (n=98) used boiled water. Age decade-wise association of the number of participants and methods of water purification is shown in Figure 1.

**Figure 2:**
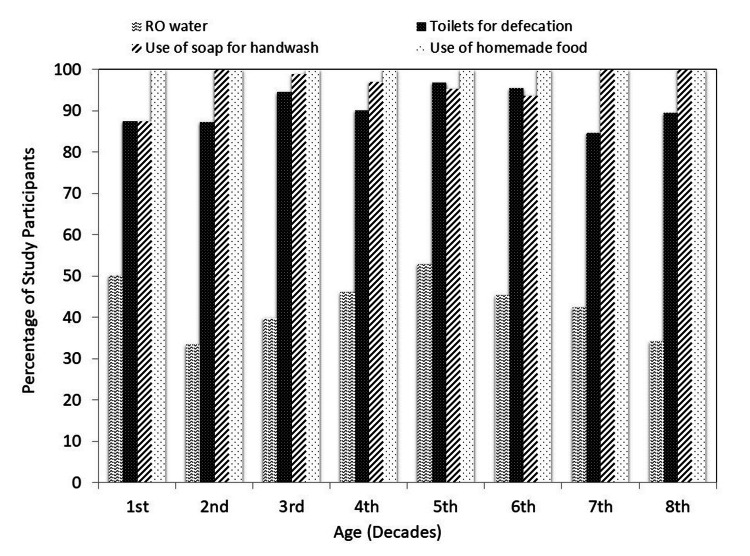
Distribution of predisposing factors RO water: reverse osmosis method of water purification

In the current study, 90.8% of the study population reported the use of toilets for defecation, and 96.7% of the cases use soap for handwash after defecation. Most of the study participants consumed homemade food. The association of the selected study variables (predisposing factors) in percentage with studied age brackets is shown in Figure 2.

Of the 1,250 study participants tested, anti-HAV IgG antibodies were detected in 1,202 (96.2%) samples. Only 48 participants tested negative for anti-HAV IgG (27 children, 20 adults, and one old). Table 3 shows the distributions according to the age groups studied. Seropositivity for anti-HAV IgG was found to be >90% in adults and >99% in old age participants; however, it was lower (79.1%) in children (one to <18 years) in the current study. No significant differences were observed in the seropositivity rates and socioeconomic class of the study participants; 89.4.4% seropositivity was observed in the upper middle class (modified Kuppuswamy score: 16-25) and 91.6% positivity in the lower upper class (modified Kuppuswamy score: 5-10).

**Table 3:** Anti‑hepatitis A virus IgG positivity in the study participants of various age groups HAV: hepatitis A virus, IgG: immunoglobulin G

Study participants	Age bracket (years)	IgG HAV status		Total
		Positive (n (%))	Negative (n (%))	
Children (1 to <18 years) (N=129)	First decade (1-10)	38 (73)	14 (37)	52
	Second decade (>10-20)	64 (83)	13 (17)	77
Adult (≥18 to <60 years) (N=928)	Third decade (>20-30)	200 (92.6)	16 (7.4)	216
	Fourth decade (>30-40)	213 (99)	2 (1)	215
	Fifth decade (>40-50)	234 (99)	2 (1)	236
	Sixth decade (>50-60)	261 (100)	0	261
Old (≥60 to 80 years) (N=193)	Seventh decade (>60-70)	138 (99.3)	1 (0.7)	139
	Eighth decade (>70-80)	54 (100)	0	54
Total				1,250

## Discussion

The presence of anti-HAV (IgG or IgM) in human serum is indicative of past or present infection with hepatitis A virus (HAV) or vaccination against HAV infection. Anti-HAV IgM is detectable during the acute stage of illness, while anti-HAV IgG may be present for many years after recovery or following vaccination [6]. Before, India was considered to be hyperendemic for HAV infection; however, in recent studies, cord blood anti-HAV antibody levels in newborns have come down to 50%-60% from nearly 100% [7], with a <70% decline in the prevalence of anti-HAV antibodies among Indian adults, which remains unexposed to the HAV infection during early childhood as reported in recent years; this warrants gathering age group-specific seroprevalence data on hepatitis A infection [8].

HAV infection is closely associated with household crowding, lower social background, unsafe water supplies, inadequate sanitation, and poor personal hygiene [9]. Acharya et al. [10] and Aggarwal et al. [11] reported that 90% of Indian children produce defensive antibodies against HAV by the age of 10 years in their respective studies on school-going children of Delhi (aged 10-17 years) and Lucknow (aged 6-10 years). Accordingly, 80% of children had anti-HAV IgG antibodies in the present study, reflecting that the remaining 20% are not exposed to HAV presumably because of better living conditions such as the availability of safe drinking water and improved sanitation and hygiene.

An epidemiological shift in the age of acquiring hepatitis A virus (HAV) infection from early childhood to adolescence and young adulthood or between urban and rural settings among the Indian population is reported in recent years [12]. Recent reports of foodborne hepatitis outbreaks in India suggests the necessity for mass vaccination of the adult population [13]. However, 97.9% of adults and 99.5% of old participants in the present study tested seropositive for the presence of anti-HAV IgG antibodies. It means that they were exposed to the HAV virus during their life span/persistence of protective antibodies for the long term and thus developed immunity. They are not at risk of developing infection again and hence do not need to be vaccinated. Similar observations with up to 90% seropositivity were reported earlier by Tandon et al. [7] in an adult population of northern India. No significant difference was found in seropositivity rates and the socioeconomic class of the study participants. This can be explained by the fact that HAV infection is considered to be endemic in India; hence, the large percentage of the residents attain immunity through asymptomatic infection/exposure early in life [14].

Limitations of the study include hospital-based sampling instead of population-based sampling for the present seroprevalence survey and a relatively small number of children less than 10 years old (less than 52), which may not provide sufficient evidence to support universal childhood vaccination being necessary in Lucknow at this time. Such a small number does not allow for a thorough comparison of universal vaccination versus targeted vaccination for children who may be unlikely to be exposed to hepatitis A virus in infancy or early childhood.

## Conclusions

We have tried to evaluate early trends of the seroepidemiological shift in HAV exposure/infection and accordingly propose age-appropriate strategies for vaccination based on sociodemographic factors if any. We find that the majority of adult and old age participants are seropositive for anti-HAV IgG antibodies. Therefore, we are of the opinion that mass vaccination of the adult population does not seem necessary, which is opposed to the suggestion made in view of foodborne hepatitis outbreaks reported in recent years in India. We have observed only 80% seropositivity in children of age below 18 years in the current study and support the current guidelines of the Indian Academy of Pediatrics, which recommends immunization for hepatitis A vaccination at the age of 12 months. It can be included as part of routine immunization in North India.
